# Somogyi effect as the most common cause of fasting hyperglycemia in T1D patients

**DOI:** 10.1186/1758-5996-7-S1-A62

**Published:** 2015-11-11

**Authors:** Walter Jose Minicucci, Frederico Fernandes Maia, Arnaldo Moura Neto, Denise E Zantut-Wittmann

**Affiliations:** 1 UNICAMP, Campinas, Brazil

## Background

Morning hyperglycemia in patients with type 1 diabetes (T1D) may be caused by the dawn phenomenon, the Somogyi effect or poor glycemic control, diagnoses that lead to different clinical management in each case. Previous reports considered the Somogyi effect as a rare entity in clinical practice.

## Objectives

This study aimed to evaluate the frequency and characteristics of fasting hyperglycemia by continuous glucose monitoring system (CGMS) in patients with T1D.

## Materials and methods

We analyzed the glycemic profile measured by 72h-CGMS in 85 patients with T1D (29 female), resulting in 255 overnight periods. We assessed the nocturnal glycemic profile and the accuracy of the CGMS sensor per night. Fasting hyperglycemia was categorized into dawn phenomenon when there was no nocturnal hypoglycemia followed by a minimum 10mg/dL rise plasma between 4: 00h-7: 00h; Somogyi effect when nocturnal hypoglycemia (< 70mg/dL) between 0: 00h-3: 00h was followed by morning hyperglycemia and poor glycemic control when there was sustained overnight hyperglycemia. Clinical data (age, diabetes duration, insulin regime, and HbA1c values) were obtained by chart review and compared to the different causes of fasting hyperglycemia.

## Results

Mean age (SD) and disease duration of participants were 30.7 (15.6) yrs. and 15 (11.9) yrs., respectively. Mean HbA1c was 8.1% (1.97%). Mean sensor glucose value was 162.6mg/dL (46.5) and mean absolute difference (MAD%) was 10.8 (4.61; reference: <28), with a correlation coefficient (CC) of 0.92 (0.1). MAD% was similar between all nights but there was a significant difference in CC of night 1 vs. nights 2 (P=0.001) and 3 (P<0.001). Fasting hyperglycemia was observed in 82.4% of patients. Silent nocturnal hypoglycemia was observed in 61.2% of patients. The main cause of elevated morning glucose levels was Somogyi effect (60%), followed by poor glycemic control (27.1%) and dawn phenomenon (12.9%). Comparing clinical characteristics of patients with dawn phenomenon and Somogyi effect yielded no significant differences.

## Conclusions

CGMS is a valid diagnostic tool to identify the causes of fasting hyperglycemic status. Somogyi effect was the most common cause of fasting hyperglycemia in T1D patients with poor glycemic control. Therefore, assessing the occurrence of nocturnal hypoglycemia might be important before increasing overnight basal insulin dose.

